# Genetic co-regulation of neopterin and Parkinson’s disease

**DOI:** 10.1038/s41531-026-01279-x

**Published:** 2026-02-10

**Authors:** Valeria Orrù, Michele Marongiu, Maristella Steri, Mara Marongiu, Carlo Sidore, Valentina Serra, Mauro Pala, Stefania Olla, Noemi Toggia, Matteo Floris, Monia Lobina, Maria Grazia Piras, Antonella Mulas, Andrea Maschio, Mariano Dei, Marina Parolini, Cinzia Dellanoce, Alessandro Delitala, 23andMe Research Team, Stella Aslibekyan, Adam Auton, Robert K. Bell, Katelyn Kukar Bond, Zayn Cochinwala, Sayantan Das, Kahsaia de Brito, Emily DelloRusso, Chris Eijsbouts, Sarah L. Elson, Chris German, Julie M. Granka, Barry Hicks, David A. Hinds, Reza Jabal, Aly Khan, Matthew J. Kmiecik, Alan Kwong, Yanyu Liang, Keng-Han Lin, Matthew H. McIntyre, Shubham Saini, Anjali J. Shastri, Jingchunzi Shi, Suyash Shringarpure, Qiaojuan Jane Su, Vinh Tran, Joyce Y. Tung, Catherine H. Weldon, Wanwan Xu, David Schlessinger, Jonica Campolo, Marcella Devoto, Magdalena Zoledziewska, Francesco Cucca, Edoardo Fiorillo

**Affiliations:** 1 Institute for Genetic and Biomedical Research, National Research Council, 08045 Lanusei, Italy; 2 Institute for Genetic and Biomedical Research, National Research Council, 09042 Monserrato, Italy; 3 Department of Biomedical Sciences, University of Sassari, 07100 Sassari, Italy; 4 Institute of Clinical Physiology, National Research Council, ASST Grande Ospedale Metropolitano Niguarda, 20162 Milan, Italy; 5 Department of Medicine, Surgery and Pharmacy, University of Sassari, 07100 Sassari, Italy; 6 National Institute on Aging Intramural Research Program – NIH – 21224-6825, Baltimore, USA; 7 Department of Translational and Precision Medicine, Sapienza University, 00185 Rome, Italy; 8 23andMe Research Institute, 94022 Los Altos, CA USA

**Keywords:** Biomarkers, Diseases, Genetics, Neurology, Neuroscience

## Abstract

Neopterin is a pro-inflammatory molecule upregulated in several diseases; however, its role in pathophysiology is unclear and its genetic regulation is unexplored. We observed that neopterin levels increase during senescence (*P-value* = 1.88×10^*-13*^, *beta* = 0.96) and positively correlate with age-related neurodegeneration and inflammation markers. The heritability estimation of neopterin variation was 35%. We then conducted a genome-wide association study on 999 Sardinians, identifying two signals in the GTP cyclohydrolase (GCH1) gene that were suggestively associated with neopterin levels. The first signal, led by rs140884539-C (*P-value* = 7.05×10^*-08*^, *beta* = 0.59), was in strong linkage disequilibrium with variants associated with predisposition to rheumatoid arthritis, decrease in dopamine, increased levels of *GCH1* transcript, dopamine metabolites, and galectin-3. The second signal, represented by rs12323905-T (*P-value* = 8.17×10^*-08*^, *beta* = 0.30), colocalised with *GCH1* splicing and Parkinson’s disease signals. Transcriptome analysis of 605 Sardinians showed that the Parkinson’s disease-predisposing variant was significantly associated with an increase in a shorter and inactive form of GCH1, whose presence is predicted to reduce the GCH1 decamer stability. The GCH1 homo-decamer regulates neopterin and tetrahydrobiopterin production, a cofactor required for the synthesis of dopamine and serotonin. Our data motivate experimental work to test whether modulating *GCH1* expression or isoform ratio alters dopaminergic function in Parkinson’s disease models.

## Introduction

Knowledge of the genetic regulation of inflammatory marker levels, which generally increase with age and disease, has improved significantly in recent years. However, the biological mechanisms underlying their genetic associations are rarely known. The inflammation-related markers produced by immune cells include neopterin (1’, 2’, 3’-D-erythro-trihydroxypropylpterin), a member of the family of pteridines that is involved in a variety of oxidation-reduction reactions in the body. Neopterin is produced by activated monocytes, macrophages, and dendritic cells upon stimulation, mainly by IFN-gamma secreted by T lymphocytes and natural killer cells during the inflammatory response^1–3^. It derives from guanosine triphosphate (GTP) catalysis by GTP cyclohydrolase-I (GCH1), phosphatase, and superoxide hypochlorite. The latter enzyme oxidises 7,8-dihydroneopterin, a potent antioxidant and radical scavenger, into neopterin. Neopterin levels are elevated in a wide range of inflammatory conditions, including malignancies, autoimmunity, cardiovascular diseases, viral and bacterial infections, and neuroinflammation, such as Parkinson’s disease^4–8^, which is likely to protect cells from the highly oxidising environment of an inflammatory site^2^. Its levels have also been considered prognostic for the treatment of HIV and other pathologies, and an indicator of complications in allograft recipients^9,10^. Neopterin can be detected in serum, urine, saliva, synovial fluid, and cerebrospinal fluid, with a direct correlation among biological fluids^11,12^.

Currently, studies investigating age- and sex-dependent variations in neopterin levels are scarce, have been conducted on small sample sizes and have produced inconsistent results^13–17^. This is probably due to differences in biological material and detection techniques. Nevertheless, this information may contribute to our understanding of the role of neopterin in age- and sex-specific pathologies. To address this issue, we evaluated urine neopterin levels in relation to age and sex. Additionally, given its status as a marker of immunological activation and neuroinflammation, we assessed its correlation with inflammatory, haematological, and biochemical parameters, as well as six neurodegeneration molecules^18–21^.

Despite its potential role as a disease biomarker, to the best of our knowledge, only one study^22^ addressed the genetic regulation of neopterin levels, identifying a signal in the *GCH1* region associated with neopterin in chronic kidney disease patients. Apart from this manuscript^22^, published correlations between neopterin levels and diseases are based on observational studies, which do not allow for determining whether increased neopterin production is a primary cause or a secondary effect of the disease.

Here, we investigated the genetic regulation of neopterin levels by estimating its heritability, performed the first described genome-wide association study (GWAS) in a general population cohort, and linked the resulting data to Parkinson’s disease^23^.

The advantage of studying the general population genetically is that the quantitative traits measured are not affected by disease or its therapy. Therefore, any genetic overlap between a trait quantified in the general population and genetic signals identified in case-control studies establishes a genuine association between the trait (neopterin) and the disease (Parkinson’s disease).

We then evaluated the molecular and functional impact of the *GCH1* genetic signal associated with neopterin and Parkinson’s disease, suggesting the related alternative splicing of *GCH1* as a potential mechanism of predisposition. A previous study investigated the possible role of *GCH1* spliced isoforms in vitro, proposing that they are less active and stable than the full-length protein^24^. Other publications used the yeast 2-hybrid system, crystallographic structure, and computational approaches to study the full-length GCH1 protein and truncated forms^25–28^. However, to the best of our knowledge, no study has applied molecular dynamics simulation to investigate the effect of GCH1 isoforms on the stability of the GCH1 dimer and decamer.

Overall, our study helps to identify the molecular mechanisms that regulate neopterin and its role in predisposition to Parkinson’s disease.

## Results

### Age and sex tendencies of neopterin and its correlation with blood parameters

**Fig. 1:**
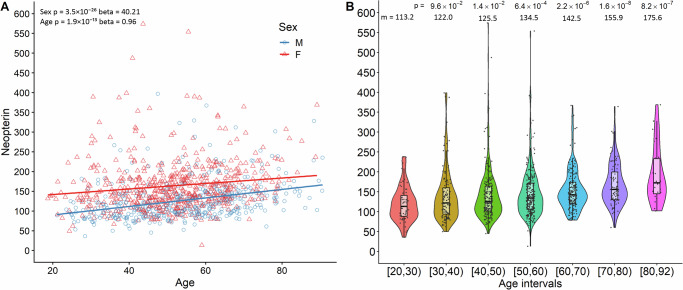
Age and sex tendency of neopterin. **A** The scatter plot represents neopterin values as a function of age, stratified by sex (males in blue and females in red). Each point represents an individual participant. The solid line represents the linear regression trend. A linear regression model was applied to calculate the effect and *P-value*. **B** The violin plot graph represents neopterin values (y-axis) stratified by age intervals (x-axis). Square brackets indicate that the age is included in the interval considered, whereas round brackets indicate that the age is not included. Internal boxplots depict the median and the interquartile range. Individual data points are shown as jittered dots. The Wilcoxon rank-sum test was used to calculate *P-values*, which are all referred to the 20–30 age range. p = *P-value*; m=median.

We measured urine neopterin-to-creatinine ratio (hereinafter reported as neopterin) in 999 general population individuals (55% females), aged 18 to 92 years, belonging to the genetically characterised SardiNIA cohort (Supplementary Data 1 and 2). The level of neopterin on average was higher in females compared to males (*P-value* = 3.51×10^-26^, *beta* = 40.21), and it increased during lifespan (*P-value* = 1.88×10^-13^, *beta* = 0.96) (Fig. 1A, B).

**Fig. 2:**
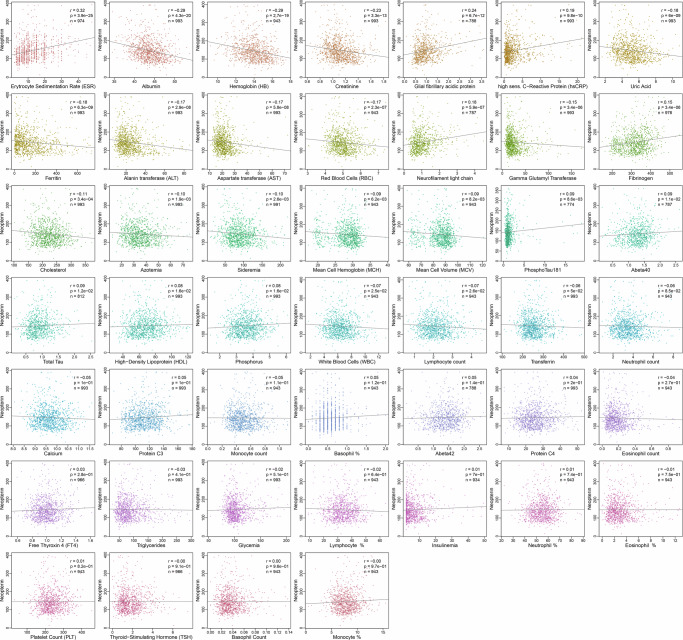
Correlation of neopterin with haematological parameters and neurodegeneration molecules. Neopterin is indicated on the y-axis and the other parameters are on the x-axis. The upper right side of each plot specifies the Spearman’s correlation coefficient (r), the corresponding *P-value* (p), and the number of individuals (n) used to calculate the correlation. The significance threshold was *P-value* = 1.09×10^-03^, obtained by dividing the nominal *P-value* of 0.05 for the number of traits assessed (*n* = 46).

To evaluate the relationship among neopterin and other disease-related markers, we also tested the correlation of urinary neopterin levels with 40 circulating haematochemical parameters and 6 neurodegeneration molecules (Fig. 2, Supplementary Data 2). As expected, neopterin positively correlated with inflammation biomarkers such as erythrocyte sedimentation rate (*P-value* = 3.91×10^-25^, *r* = 0.32), C-reactive protein (*P-value* = 9.84×10^-10^, r = 0.19), and fibrinogen (*P-value* = 3.4×10^-06^, *r* = 0.15). Ten further parameters, including albumin, haemoglobin, and ferritin, were inversely correlated with neopterin levels (Supplementary Data 2). Among the neurodegeneration molecules measured, we observed a significant positive correlation between neopterin and glial fibrillary acidic protein (*P-value* = 6.70×10^-12^, *r* = 0.24) and, to a lesser extent, between neopterin and neurofilament light-chain (*P-value* = 5.90×10^-07^, *r* = 0.18) (Supplementary Data 2).

### Fine mapping of neopterin genetic association signals in the *GCH1* locus

We first quantified the contribution of genetic factors to trait variability using variance-component models based on pedigree-derived familial relationships (see Methods). In a base model, total phenotypic variance (Var(Y)) was partitioned into a genetic component shared according to kinship (σ_g_²) and an individual-specific environmental component (σ_e_²). This yielded a ‘broad-sense heritability’ estimate (H^2^ = σ_g_^2^/Var(Y)) of 0.35, indicating that 35% of the observed variation reflects underlying genetic differences among individuals. We next fitted a refined model in which the genetic variance (σ_g_^2^) was decomposed into additive (σ_a_^2^) and dominance (σ_d_^2^) components, allowing estimation of narrow-sense heritability (h^2^ = σ_a_^2^/Var(Y)), which captures only additive effects relevant for association analyses. The resulting estimate, h² = 0.22, indicates that 22% of the trait variability is attributable to additive genetic effects. The difference between H² and h² suggests that non-additive genetic factors, including dominance and epistasis, also contribute substantially to trait variation (Supplementary Data 3).

**Fig. 3:**
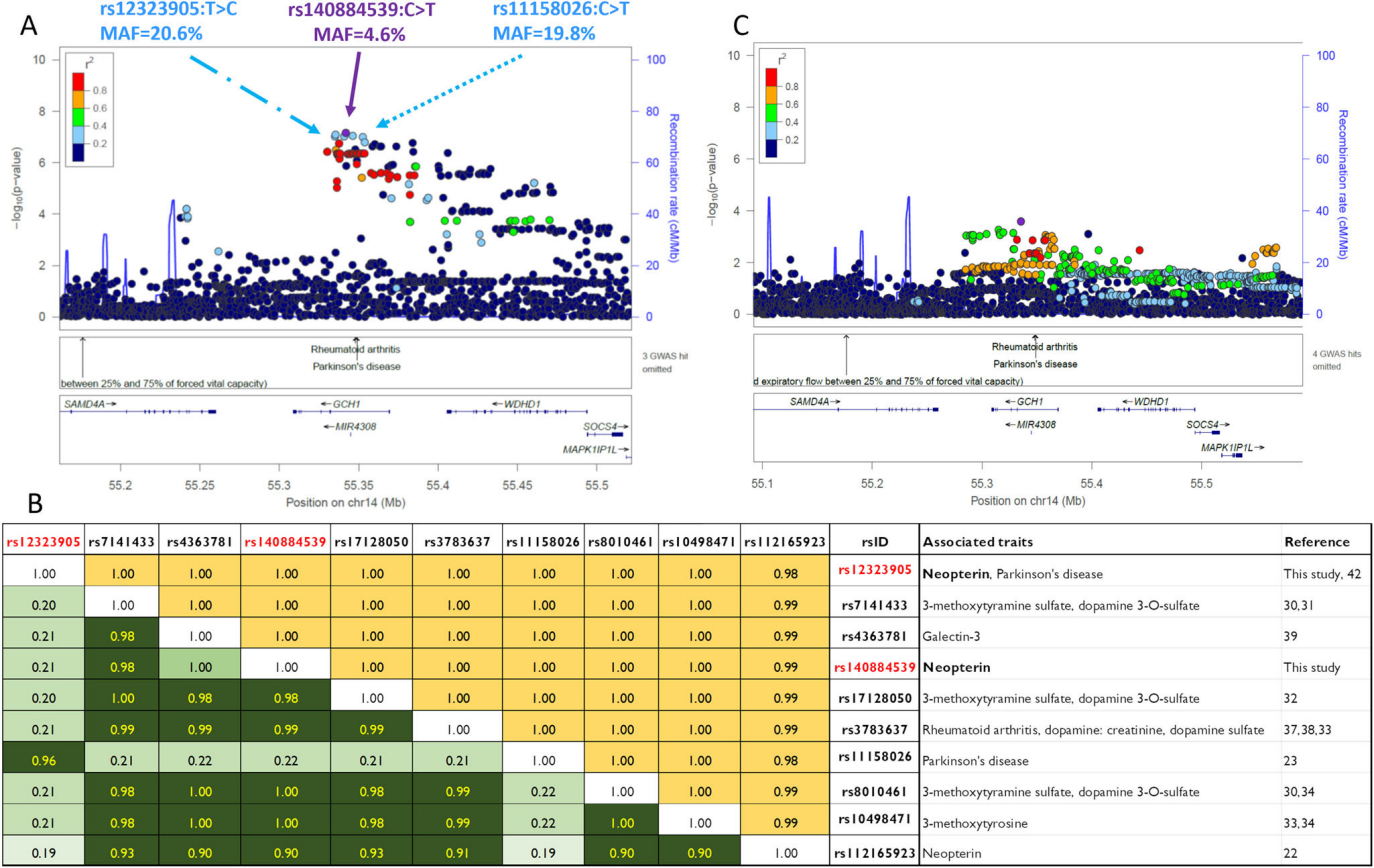
Neopterin association and conditional analysis of the *GCH1* region. Regional plots derived from **A** GWAS and **C** conditioning for variant rs140884539. The significance of the association ( − log10[P value]; left y-axis) for each trait is plotted relative to the genomic positions on the hg19/GRCh37 genomic build (x-axis). SNPs are coloured to reflect their linkage disequilibrium with the top SNP (indicated with a purple dot and its genomic position at the built GRCh37/hg19). **B** Linkage disequilibrium among SNPs in the *GCH1* gene region associated with quantitative traits and/or diseases. The reference population is Europeans from the 1000 Genome project (https://www.internationalgenome.org/). Linkage disequilibrium was calculated by the National Institute on Health LDtool (https://ldlink.nih.gov/?tab=ldmatrix). r^2^ is indicated in green, D’ in yellow. The strength of correlation is colour-coded: the higher the correlation, the darker the colour.

We thus explore the genetic regulation of neopterin by performing the first GWAS conducted in a general population cohort. We observed suggestive associations of neopterin levels within the *GCH1* locus (Fig. 3A, Supplementary Data 4). The protein encoded by this gene (GTP Cyclohydrolase 1) is involved in the enzymatic process that generates neopterin from GTP. This enzyme also participates in tetrahydrobiopterin (BH4) production, a cofactor necessary for nitric oxide, dopamine, and serotonin biosynthesis^29^.

The most associated variant with urine neopterin levels was the intronic single-nucleotide polymorphism rs140884539, with major allele rs140884539-C positively associated with the trait (*P-value* = 7.05 × 10^-08^, *beta* = 0.59).

The variant rs140884539 is in linkage disequilibrium (LD) (r^2^ = 0.90) with another intronic polymorphism in *GCH1*, rs112165923, already reported to be associated with neopterin levels in a previous GWAS on a chronic kidney disease cohort^22^ (Fig. 3B). The allele rs140884539-C is also in strong LD (r^2^ > 0.98) with allelic variants increasing the levels of dopamine 3-O-sulphate and of 3-methoxytyramine, two dopamine metabolites^30–34^, and of 3-methoxytyrosine^31,35^, an active and potentially toxic metabolite of L-DOPA associated with Aromatic L-amino acid decarboxylase deficiency^36^ (Fig. 3B). Furthermore, rs140884539-C is in strong LD (r^2^ > 0.99) with variants associated with rheumatoid arthritis predisposition^37^, dopamine level decrease^38^, and galectin-3 increase^39^ (Fig. 3B). The latter protein is known to be involved in inflammatory responses^40,41^.

To search for further genetic signals associated with neopterin that are potentially hidden by the strongest signal, but independent from it, we performed a conditional analysis. This analysis statistically removed the association of the lead variant rs140884539 and allowed the identification of a second suggestive signal in the *GCH1* gene region (Fig. 3C, Supplementary Data 4).

To better evaluate the number of signals associated with neopterin in the *GCH1* gene region, we performed a fine-mapping analysis (Methods). The probability of having two independent neopterin signals in *GCH1* was 60% against 39% of having only one signal. To prioritise the neopterin-associated variants of the two signals in the *GCH1* region, we identified the group of variants having a 95% posterior probability of being causal (also known as the credible set). The credible set for the first signal included 33 variants, with the most probable being rs140884539 (Supplementary Data 5). The credible set of the second signal comprised 1439 variants, all of which had a small posterior probability of being causal. This second credible set included rs12323905 and rs11158026, associated with Parkinson’s disease in a case-control study^42^ and in large European meta-analyses^23,43,44^, respectively (Fig. 3B, Supplementary Fig. 1). Furthermore, rs12323905 was among the most probable variants to be causal, with a higher posterior probability than rs11158026 (Supplementary Data 6), and was the second most associated variant in our neopterin GWAS (*P-value* = 8.17×10^-08^, *beta* = 0.299, in the discovery analysis); thus, we selected rs12323905 as representative of the second neopterin signal.

The two prioritised variants, rs140884539 and rs12323905, have very different minor allele frequencies (MAF = 4.6% and 20.6%, respectively) in the SardiNIA cohort and are in weak LD with each other (r² = 0.21). Specifically, allele rs140884539-C (AF = 95.4%) was found on haplotypes containing both T (78%) and C (16%) of rs12323905, whereas rs140884539-T was found associated only with rs12323905-C (4.6%) (D’ = 1) (Supplementary Fig. 1). The statistical power to detect the effects observed for the 2 prioritised variants was 75% for rs140884539 and 67% for rs12323905 in a sample size of 999 individuals (see Methos for details).

To further evaluate the relative contribution of rs140884539 and rs12323905 in the neopterin association, we performed a combined analysis, with rs140884539 having twice the effect of rs12323905 in regulating the neopterin level. However, the contribution of the two genetic variants tested was modest, accounting for approximately 3% of the variation in neopterin levels, whereas sex and age accounted for 13% and 6%, respectively (Supplementary Data 7).

Additionally, gene set enrichment analysis, using the Enrichr tool (https://maayanlab.cloud/Enrichr/) and the 32 gene regions suggestively associated with neopterin levels in our GWAS data (listed in Supplementary Data 4), showed that Parkinson’s disease and BH4 synthesis are primarily linked to neopterin-associated genes (see Supplementary Fig. 2).

Overall, these results suggest a common genetic regulation between neopterin and Parkinson’s disease.

### Transcriptional profiling of the *GCH1* gene and functional implications

**Fig. 4:**
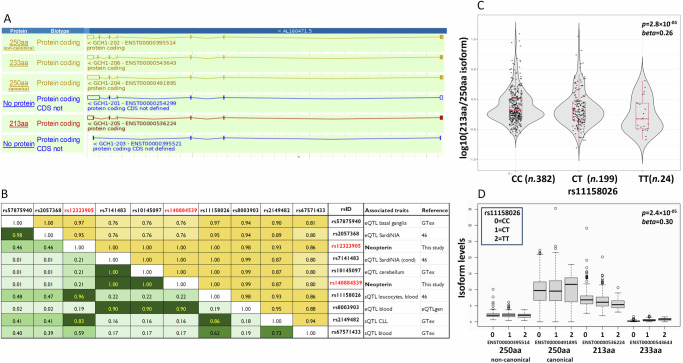
*GCH1* isoform expression stratified by rs11158026 genotypes. **A** *GCH1* transcripts from Ensembl genome https://www.ensembl.org. **B** Linkage disequilibrium among SNPs in the *GCH1* gene region associated with neopterin, eQTL and sQTL data. Specifications are as in Fig. 3B. **C** The violin plot illustrates the ratio of the 213aa to the canonical 250aa isoforms. **D** The box plot graph shows the levels of the *GCH1* isoforms described in (**A**). The reported *P-values* and effects refer to rs11158026-C and are calculated by applying a linear mixed model.

The *GCH1* gene transcript is characterised by six isoforms comprising two full-length forms differing in the 3’UTR region and encoding a 250 amino acids (aa) protein, two mRNA splice variants encoding 213 and 233 residue proteins with alternative C-termini^45^, and two non-coding isoforms (Fig. 4A). A further potential isoform has been recently predicted (UCSC 10-11-23 release, NM_001424105.1).

We thus focused on the transcriptional analysis of *GCH1* gene variants using expression quantitative trait loci (eQTLs) and splicing QTLs (sQTL) from the SardiNIA cohort^46^, GTEx, and eQTLgen databases (https://gtexportal.org/ and https://www.eqtlgen.org/).

Transcriptome analysis on leucocyte data from 605 SardiNIA individuals detected two eQTL signals in the *GCH1* gene region (Supplementary Data 8). The variant most associated with *GCH1* expression in the SardiNIA data, rs2057368, was in strong LD (r^2^ = 0.98) with the most associated eQTL signal in basal ganglia, which plays a key role in dopamine production (Fig. 4B). After conditioning on rs2057368, a further eQTL signal, led by rs7141483, was detected. Variant rs7141483 was in perfect LD (r^2^ = 1) with rs140884539 associated with neopterin (Fig. 4B), with alleles associated with increased neopterin also augmenting *GCH1* expression.

Furthermore, in the SardiNIA dataset, we found that the regulatory variant rs11158026, associated with Parkinson’s disease^23,44^ and in strong LD with rs12323905 (r2 = 0.96, Fig. 4B), was an sQTL for *GCH1*. The Parkinson’s disease predisposing allele rs11158026-C was associated with a significant increase in the 213aa-250aa *GCH1* isoform ratio (Fig. 4C, effect of rs11158026-C, *P-value* = 2.8×10^-04^, *beta* = 0.26), primarily due to an increase in the 213aa isoform (Fig. 4D, effect of rs11158026-C, *P-value* = 2.4×10^-05^, *beta* = 0.30), while the 250aa canonical isoform showed a non-significant downward trend (Fig. 4D, effect rs11158026-C, beta = -0.05, p-value = 0.4). The other two isoforms detected (non-canonical 250aa and 233aa long) were weakly expressed (Fig. 4D). Notably, the canonical and the 213aa isoforms are known to interact with each other, producing GCH1 decamers that were proposed to be less active and stable than those formed by the full-length proteins only^24^.

Interrogating public databases such as eQTLgen and GTex, we observed that variant rs140884539 is in LD (r^2^ = 0.90) with the most associated eQTL variant in blood (eQTLgen) and in perfect LD (r^2^ = 1) with the most associated eQTL variant in the cerebellar hemisphere (GTex and Klein et al., 2023)^47^ (Fig. 4B, Supplementary Data 9). Furthermore, rs12323905 is in LD (r^2^ = 0.83) with the most associated sQTL variant in lymphoblastoid cell lines in GTex (Fig. 4B, Supplementary Data 10).

Overall, these data support the role of the first neopterin signal in regulating *GCH1* expression and of the second signal in regulating *GCH1* splicing events.

### Colocalisation analysis

**Fig. 5:**
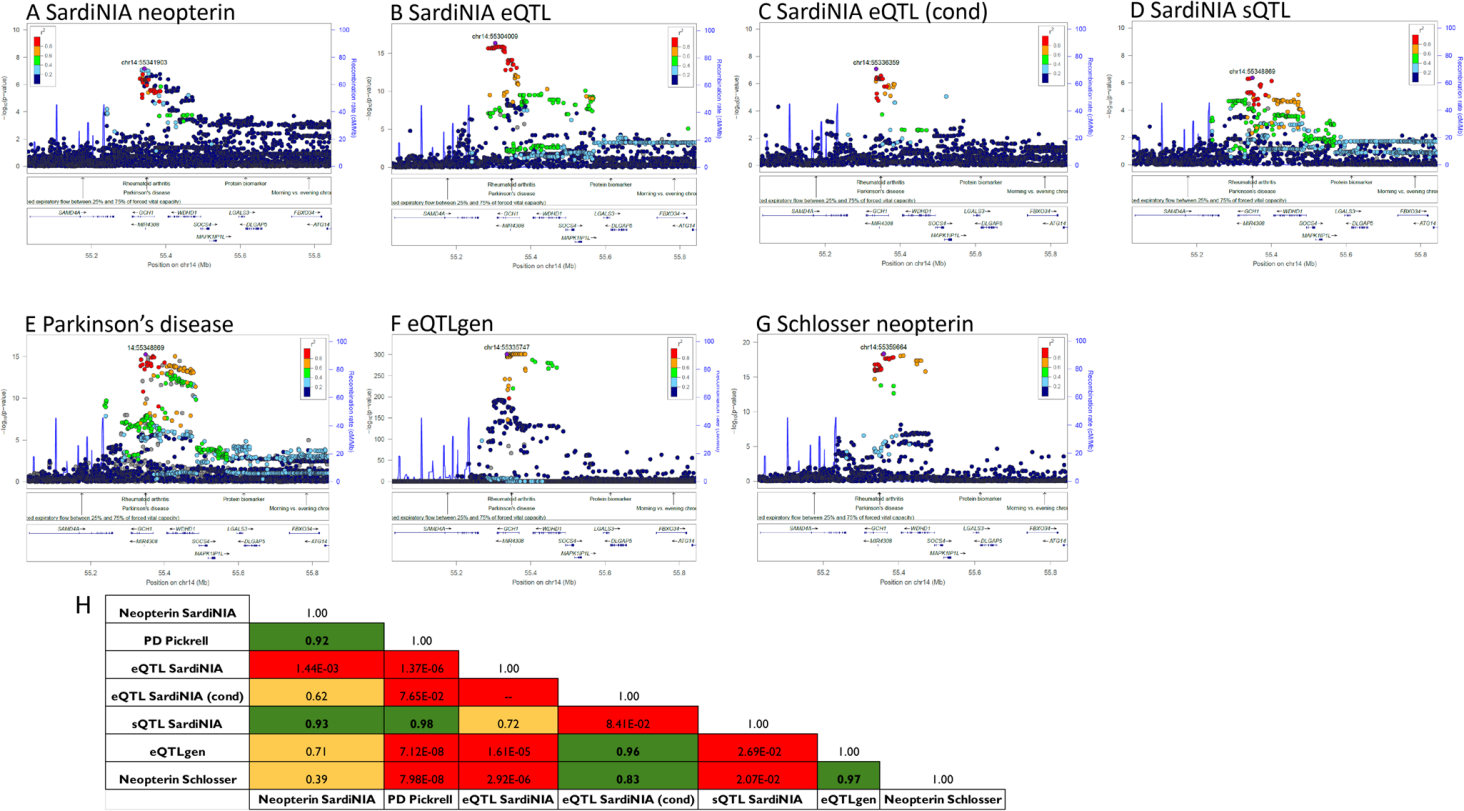
Colocalisation among neopterin, Parkinson’s disease, *GCH1* eQTL, and sQTL signals. Regional association plots of **A** SardiNIA neopterin, **B** SardiNIA eQTL, **C** SardiNIA eQTL after conditional analysis, **D** SardiNIA sQTL of ENST00000536224, **E** Parkinson’s disease, **F** *GCH1* eQTL from eQTLgen database, and **G** neopterin by Schlosser et al. ^22^. The regional plot description is as in Fig. 3. Parkinson’s disease-associated variants were kindly shared by 23andMe (https://research.23andme.com/dataset-access/, Pickrell dataset). **H** The posterior probability of two traits sharing a single causal variant (H4) is reported in each quadrant. The quadrant is in green if H4 indicates colocalisation, it is in yellow if the colocalisation is uncertain, and in red if there is no colocalisation (ns = not significant).

To better understand the role of neopterin and *GCH1* in Parkinson’s disease risk, we performed colocalisation analysis among neopterin, Parkinson’s disease^23^, and SardiNIA QTL data in the *GCH1* gene region (Fig. 5). SardiNIA neopterin association colocalised with Parkinson’s disease (*H4* = 0.92) and *GCH1* sQTL (H4 = 0.93) signals. Furthermore, Parkinson’s disease colocalised with the *GCH1* sQTL (H4 = 0.98), but not with *GCH1* eQTL signals.

Further colocalisations between neopterin and other published data, and the related regional plots are reported in Fig. 5 and Supplementary Data 11.

### Interaction between the 250aa and 213aa GCH1 proteins

**Fig. 6:**
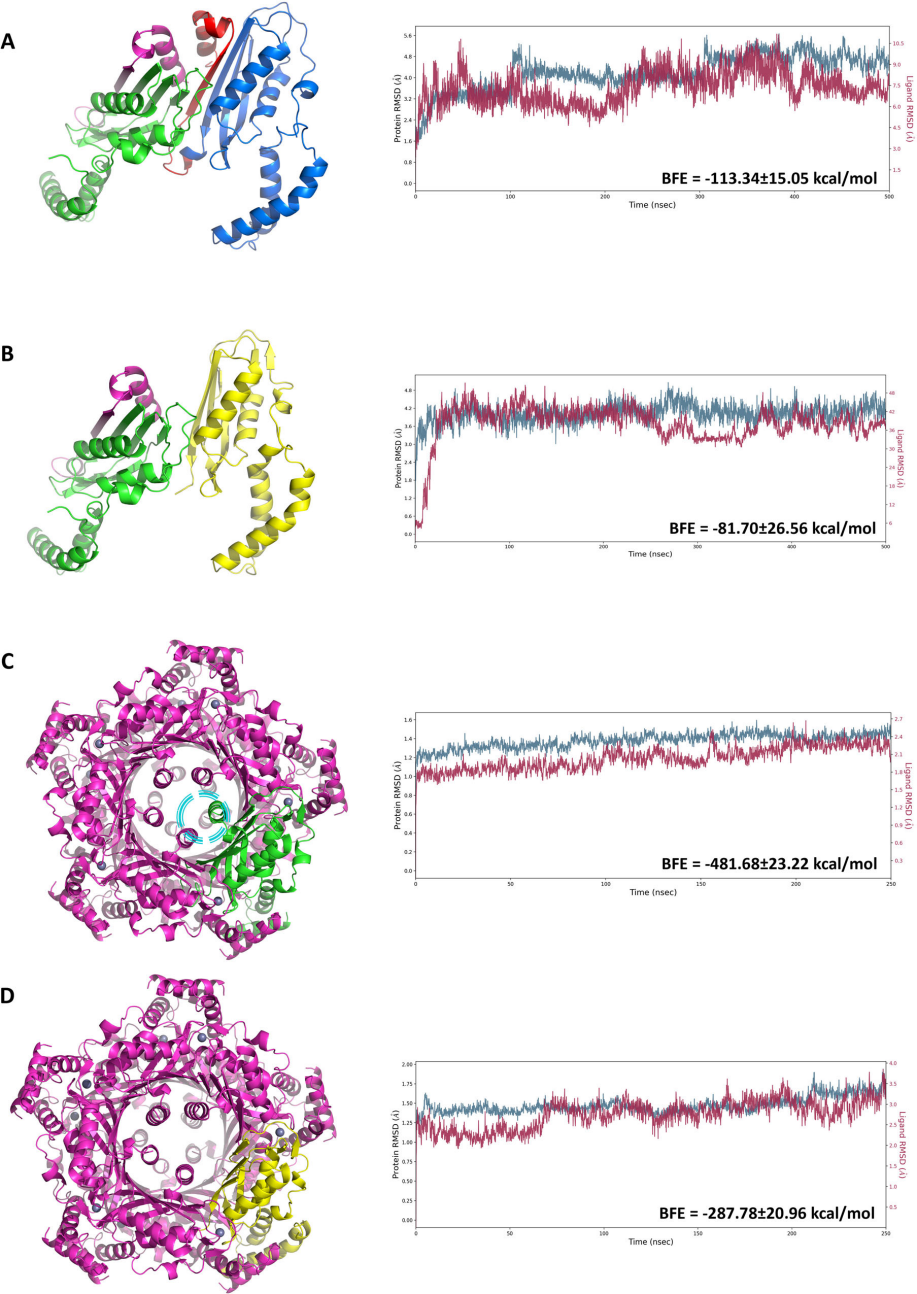
3D representation and molecular dynamics simulations of the wild-type and mutated GCH1 dimers and decamers. The crystallographic structures are reported on the left and the corresponding molecular dynamics simulation on the right. **A** Wild-type dimer is shown with the two 250aa chains coloured in green and blue. Residues absent in the 213aa isoform are highlighted in magenta and red. **B** Mutated dimer is displayed and the 213aa short isoform is coloured in yellow. **C** Wild-type decamer, one chain is shown in green. The region corresponding to the residues absent in the short isoform, and involved in the formation of the catalytic site, is indicated by the light blue circle. **D** Mutated decamer with the 213aa chain represented in yellow. In each graph on the right, the x-axis represents the simulation time expressed in nanoseconds (nsec), while the y-axis shows the root mean square deviation (RMSD) expressed in Ångströms (Å). The violet line corresponds to the monomer used as the ligand within the complexes: this refers to the 250aa chain in the wild-type forms and to the shorter 213aa isoform in the mutated complexes. The blue line represents the RMSD of the remaining protein chains. BFE=binding free energy. The crystallographic structures of the wild-type GCH1 dimer and decamer are from https://www.rcsb.org/structure/1FB1andhttps://www.rcsb.org/structure/6Z80. The 213aa isoform was predicted by AlphaFold2 software. The stability of the GCH1 dimer and decamer was evaluated by Desmond software.

To evaluate the stability of the dimer and decamer of GCH1 in the presence of the 213aa short isoform, we generated the corresponding three-dimensional model (see Methods for details). Then, we modelled the wild-type and mutated dimers and decamers, both containing a single mutated chain (Fig. 6). We initially evaluated the stability of the different GCH1 forms through molecular dynamics simulations, performing a total of four simulations: two 500nsec simulations for the wild-type and mutated dimers (the first composed of two 250aa chains and the second of one 250aa chain and one 213aa chain) (Fig.6A, B), and two 250nsec simulations for the wild-type and mutated decamers (the latter including a single 213aa chain) (Fig. 6C, D). Our results showed that both wild-type forms—the dimer and the decamer—are more stable than their corresponding mutated versions. This observation was further supported by Molecular Mechanics/Generalised Born Surface Area (MMGBSA) analysis. Specifically, the binding free energy for the mutated dimer was −81.70 ± 26.56 kcal/mol compared to −113.34 ± 15.05 kcal/mol for the wild-type dimer. Considering the decamers, the mutated form displayed a binding free energy of −287.78 ± 20.96 kcal/mol, while the corresponding wild-type value was -481.68 ± 23.22 kcal/mol; the latter was more negative, indicating a more favourable binding energy.

Supplementary Results are available in the Supplementary Information.

## Discussion

In the present work, we cross-sectionally evaluated neopterin levels throughout the lifespan. It varies by 0.96 units per year of age, with an approximate increase of 50% in people over 70 compared to those in their 20 s (five decades). This change in neopterin levels represents a physiological increase mainly due to the basal inflammatory state typical of the elderly, known as inflammaging. Notably, the neopterin increase is much more pronounced in acute and severe infections (such as tuberculosis, Malaria, COVID)^48–52^, in which it reflects the body’s immediate, strong reaction to a pathogen.

We also correlated neopterin levels with 46 proteins and molecules circulating in the blood. We observed that parameters that correlated positively with neopterin (such as neurodegeneration and inflammation markers) increase with senescence, supporting the role of neopterin in neuroinflammation. Conversely, the parameters that correlated negatively with neopterin (such as haemoglobin iron and albumin) generally decrease with ageing.

**Fig. 7:**
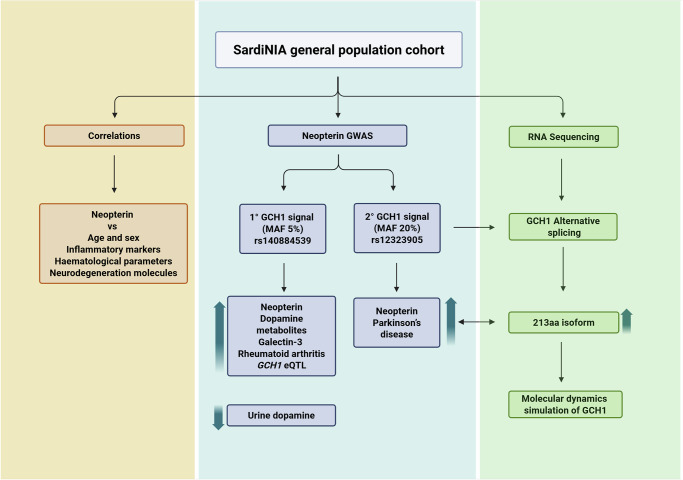
Workflow and main considerations of the study. (Left, orange) Correlation results showed a positive relationship between neopterin and age, as well as markers of inflammation and neurodegeneration. (Middle, blue) GWAS results support the presence of two signals in the *GCH1* region associated with neopterin levels. These signals are also associated with pathologies and metabolites. (Right, green) RNA sequencing data support the presence of a *GCH1* alternative splicing transcript of 213aa. Molecular dynamics simulations predict the dimerisation and decamerisation of the 213aa isoform with the wild-type chain, as well as a reduced stability of the complexes containing the short protein. Created in BioRender. Fiorillo, E. (2025) https://BioRender.com/o8gxd2v.

We also identified a link between neopterin and Parkinson’s disease *via* genetic analyses and proposed a potential role for the neopterin pathway in disease predisposition (Fig. 7). Importantly, the genetic analysis of neopterin was conducted in a general population cohort that is free of a potential “reverse causality” bias due to the presence of the disease or its treatment with drugs. Thus, neopterin association with the *GCH1* gene cannot be due to Parkinson’s disease because detected in the general population; rather, the disease risk might be partially mediated by the neopterin pathway.

Genetic regulation accounts for 35% of neopterin variability. This variability drops to 22% when considering only the additive genetic effect, with variants in *GCH1* contributing 3%. Although common variants typically have modest individual effects on quantitative traits, their aggregate additive contributions ultimately shape overall trait levels^53^. Consistent with this, the effect of variants in *GCH1* on trait regulation and on Parkinson’s disease susceptibility (OR = 1.1)^43^ is modest, but it may become significant when combined with other predisposing genetic risk factors and/or relevant environmental exposures.

We detected two neopterin genetic signals in the *GCH1* region. The first signal was also associated with *GCH1* eQTL, whereas the second signal was associated with *GCH1* splicing events. Notably, the variant most associated with Parkinson’s disease was included in the second signal and was an sQTL in SardiNIA transcript data.

These data, along with the lack of colocalisation between the *GCH1* eQTL signal and Parkinson’s disease signal, support *GCH1* alternative splicing as the main molecular mechanism associated with Parkinson’s disease risk in the *GCH1* region. Our results support the hypothesis that at basal and peripheral levels, the second neopterin signal, which increases neopterin and predisposes to Parkinson’s disease, is associated with an increase in the 213aa and catalytically inactive isoform of GCH1. Molecular dynamics simulations predicted that the presence of 213aa monomers reduced the GCH1 decamer stability. This short isoform is characterised by the absence of residues involved in binding of the GCH1 feedback regulator (GCHFR) and was proposed to fine-tune the GCH1 function^24^, favouring neopterin production following specific insults.

Indeed, after stimulation of monocytes with IFN-gamma, GCH1 production increases up to 100-fold, and the long and active form is produced in larger amounts than the short form, overall increasing the GCH1 enzymatic activity and neopterin production^54^. Thus, splice variants were proposed to regulate GCH1 by reducing its basal activity and allowing a broader dynamic range after induction in a tissue-specific and probably insult-specific manner^55^.

**Fig. 8:**
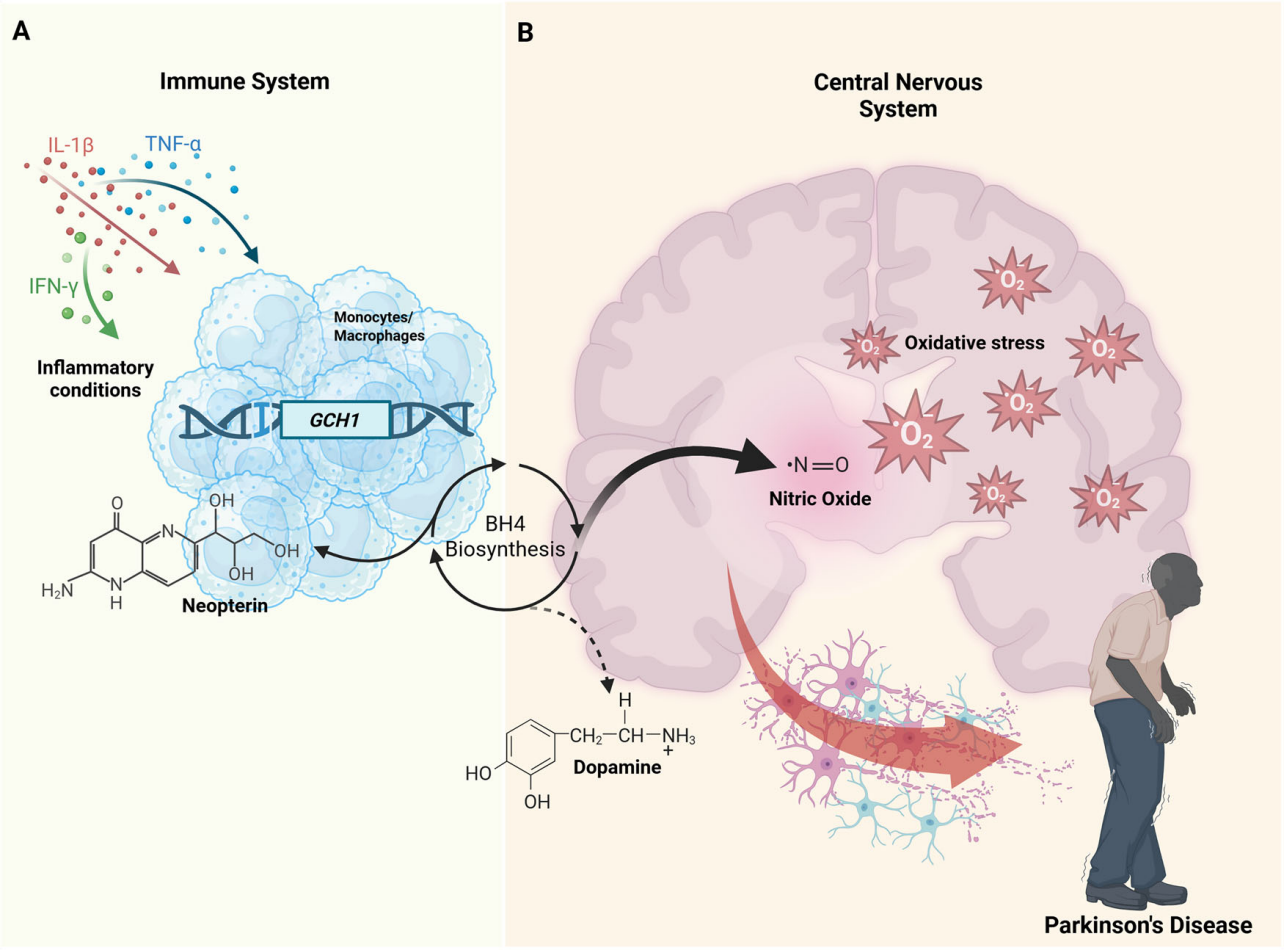
Neopterin association with Parkinson’s disease pathways. The figure illustrates the role of neopterin within the main pathways that predispose to Parkinson’s disease. Panel **A** shows the events leading to an increase in peripheral neopterin levels. Under inflammatory conditions, monocytes/macrophages enhance neopterin production as a by-product of tetrahydrobiopterin (BH4). Panel **B** shows the impact of inflammation on neopterin production in the central nervous system, with increased neopterin levels not accompanied by a similar increase in BH4. Consequently, dopamine production is impaired, resulting in the preferential formation of nitric oxide and causing neuronal damage. Created in BioRender. Fiorillo, E. (2025) https://BioRender.com/kfzgzuw.

To add an extra layer of complexity, GCH1 is not only involved in neopterin production by the immune system, but it also generates nitric oxide in the vessel endothelium and catalyses dopamine, serotonin, and nitric oxide production in neurons (Fig. 8). All these pathways play a key role in Parkinson’s disease risk and may be genetically regulated in a specific manner. In this scenario, the presence of short isoforms of GCH1, capable of assembling with the long form, might have different implications for the regulation of catalytic activity, likely reducing the function of the decameric forms in the brain, but not in the immune system. In fact, in inflammatory conditions, increased production of GCH1 results in elevated levels of neopterin not accompanied by a similar BH4 increase. This occurs because downstream enzymes, such as 6-pyruvoyltetrahydrobiopterin synthase, implicated in BH4 production, do not significantly rise, thereby becoming the limiting factor for BH4 synthesis in immune cells^54^. In neurons, BH4 is a key cofactor in the production of both nitric oxide and dopamine and has a much higher affinity for nitric oxide synthase (NOS) than tyrosine hydroxylase (TH), the latter involved in dopamine production^54^. Therefore, under inflammatory conditions where high nitric oxide production is required, BH4 is primarily engaged in NO compared to dopamine production and is more oxidised due to more reactive oxygen scavengers, further reducing the levels available for dopamine production.

This might explain, at least in part, the presence of increased neopterin in the periphery, accompanied by reduced BH4 and neurotransmitters in the brain in neurodegenerative pathologies, such as Parkinson’s disease^54,56^.

An inverse correlation between neopterin and BH4 was also observed during senescence^57^ and in pathological conditions^58,59^ and might partially explain the basal inflammation observed in the elderly, known as inflammaging^60^, along with reduced production of neurotransmitters typical of neurodegeneration^61^.

In line with this scenario, *Gch1*-deficient mice have low brain levels of BH4, catecholamines, serotonin, and metabolites, which align with the neurochemical findings in human patients with mutations in the *GCH1* gene. Indeed, mutations in the *GCH1* gene are known to cause DOPA-responsive dystonia and BH4-deficient hyperphenylalaninemia^62^

Consistent with our observations, gene transfer of the enzymes central for dopamine synthesis, such as tyrosine hydroxylase (TH) and GCH1, has been considered a potential therapeutic strategy to enhance production of DOPA in the brain striatum. This notion was confirmed in preclinical studies in a rat model of Parkinson’s disease^63,64^. More recently, clinical trials consisting of using viral vectors to deliver DNA coding for one or more genes involved in Parkinson’s disease pathogenic mechanisms, such as key enzymes responsible for dopamine production (i.e., TH, GCH1, and AADC), were considered an alternative to increase the bioavailability of dopamine in the nigrostriatal synapses^65^.

Our study has several limitations. Indeed, future parallel measurement of neopterin and BH4 (as well as dopamine, and its metabolites) in larger cohorts of genetically characterised individuals in health and disease could help clarify the relationship between the two pteridines at phenotypic and genetic levels and their involvement in neurodegeneration, inflammatory diseases, and senescence. This will lead to a better understanding of the biological mechanisms underlying neurodegeneration and the identification of more specific therapeutic targets.

Furthermore, studying the immune response in individuals with different *GCH1* genotypes at baseline and after immune stimulation would enable us to evaluate how genetics may affect the expression of *GCH1* isoforms and their downstream metabolites in response to external insults. However, some of these metabolites, especially BH4 and dopamine, are rarely detectable in physiological conditions because they are very unstable and easily perishable, making them difficult to measure, especially from frozen samples that would require specific pre-treatment for reliable quantification.

Still, the genetic data observed in the study are suggestive. Thus, increasing the sample size would improve our ability to detect genetic associations, enabling us to discriminate the identified signals more effectively. This would strengthen our findings and reveal further associations, which are necessary for applying statistical approaches such as Mendelian randomisation to test the causal role of neopterin in Parkinson’s disease. Notably, as Parkinson’s disease is highly heterogeneous in terms of its clinical manifestations, progression and treatment response^66,67^, we cannot rule out the possibility that its association with GCH1 may be specific to a particular clinical subtype. Thus, careful clinical characterisation of patients with Parkinson’s disease could help us to understand the genetic contribution to disease predisposition better. Finally, the stability of GCH1 isoforms predicted by molecular dynamics is a computational inference that needs to be confirmed by functional studies in vitro and in vivo. Therefore, the absence of functional studies can be considered a further limitation of this work.

To our knowledge, this is the first study that proposes a genetic link between neopterin and Parkinson’s disease using agnostic statistical approaches. Our data support the beneficial effect of implementing GCH1 in the treatment of Parkinson’s disease and suggest a potential GCH1 mechanism that predisposes to the disease.

## Methods

### The SardiNIA dataset

The SardiNIA project is a longitudinal study comprising 8000 general population individuals, native to the central-east coast of Sardinia, Italy^68^. All volunteers are deeply genetically and phenotypically characterised, and 999 of them, randomly selected, were evaluated for urine neopterin levels in the present study. All participants signed informed consent to study protocols approved by the Sardinian Regional Ethics Committee (prot. n. 2171/CE).

### Neopterin measurement

Urine samples were collected from each participant once, along with blood samples for biochemical assessment, in the mornings from 2017 to 2019. Urine samples were stored at −20 °C, thawed and centrifuged at 4000 rpm for 15 min. Supernatants were then adequately diluted with a chromatographic mobile phase consisting of 15 mM K2HPO4, pH 3 to measure neopterin concentrations by isocratic high-pressure liquid chromatography (HPLC). To account for urine concentration levels, we used creatinine levels to normalise the neopterin measurements and obtain the neopterin-to-creatinine ratio^14^, which is the trait used for genetic and phenotypic analysis. Neopterin and creatinine separations were performed using a 5-μm Discovery C-18 analytical column (250 × 4.6 mm I.D) at 50 °C and at flow rate of 0.9 mL/min on a ProStar Varian ProStar HPLC system coupled to a fluorimetric detector (JASCO FP-4025, λex = 355 nm; λem = 450 nm) for neopterin assessment and to an UV–VIS detector (BIO-RAD SPD 10AV, λ = 235 nm) for creatinine determination.

### Measurement of haematochemical parameters

The 999 SardiNIA individuals evaluated for neopterin were simultaneously assessed for 40 haematological and biochemical parameters (Supplementary Data 1). Sixteen haematological parameters were measured by the UniCel DxH Coulter Cellular Analysis System (Beckman Coulter) according to the manufacturer’s instructions and using blood collected in EDTA tubes. Erythrocyte Sedimentation Rate (ESR) was measured from blood collected in sodium citrate tubes by the KIMAsed rack 10 (VacuTest Kima,#K142600, Italy). Fibrinogen was measured by Coa Data 2001 analyser (Helena Biosciences, Europe) with the Clauss Fibrinogen 100 kit (Helena Biosciences Europe, #5376) starting from plasma collected in sodium citrate tubes.

Sera isolated from blood collected in clot activator tubes (vacutest,#11030) were used to evaluate 22 biochemical parameters, of which 19 were measured by the BA200 Life Technology analyser (BioSystems). Three, including Free Thyroxin 4 (FT4), insulinemia, and Thyroid-Stimulating Hormone (TSH), were measured by the Cobas E411 analyser (Roche), following the manufacturer’s instructions (Supplementary Data 1).

### Measurement of neurodegeneration molecules

Plasma samples from 5204 SardiNIA participants were isolated from sodium citrate blood tubes and measured for six neurodegeneration molecules: Tau, phosphoTau-181, amyloid beta 40 and 42, neurofilament light chain, and glial fibrillary acidic protein (Supplementary Data 1) by Quanterix HD-X analyser following manufacturer instructions. To evaluate the phenotypic correlation between urine neopterin levels and the six circulating molecules, urine and plasma samples were collected from the same SardiNIA volunteers.

### Impact of age and sex on urine neopterin and correlation with haematological parameters

To test the impact of age and sex on urine neopterin, a linear regression model, including both variables as predictors, was applied. To correlate urine neopterin with serum haematological parameters (listed in Supplementary Data 1), Spearman’s correlation coefficient was calculated. The significance threshold of *P-value* = 1.09 × 10^-03^ was obtained by dividing the nominal *P-value* of 0.05 for the number of traits assessed (*n* = 46). Analyses were performed using R software version 4.2.3.

### Heritability estimation of urine neopterin levels

Heritability was estimated using variance-component models implemented in poly-0.5.1 software, as previously described^68^. In the base model, phenotypic variance (Var(Y)) was partitioned into a polygenic component (σ_g_²) and an environmental component (σ_e_²). The environmental variance was modelled as individual-specific, whereas the polygenic variance was shared between individuals in proportion to their kinship coefficient (φ_ij_), such that Var(Y)=σ_g_^2^ + σ_e_^2^ and Cov(Y_i_,Y_j_)=2ϕ_ij_σ_g_^2^. Broad-sense heritability was defined as H^2^ = σ_g_^2^/Var(Y).

In the refined model, genetic variance (σ_g_²) was decomposed into additive (σ_a_²) and dominance (σ_d_²) components using generalised kinship coefficients to derive Δ_ij_, the probability that individuals share both alleles identical by descent. Variance and covariance were specified as Var(Y)=σ_a_^2^ + σ_d_^2^ + σ_e_^2^ and Cov(Y_i_,Y_j_)=2ϕ_ij_σ_a_^2^+Δ_ij_σ_d_^2^. Narrow-sense heritability was estimated as h^2^ = σ_a_^2^/Var(Y).

The two models, adjusted for age and sex, were fitted by maximising the multivariate normal likelihood, with urine neopterin levels normalised using the inverse normal transformation before analysis.

### Genotyping and imputation

Genetic analyses were performed on 999 samples genotyped with the OmniExpress Illumina array as previously described for the entire SardiNIA cohort^69^. More in detail, individuals and SNPs were included in the analyses with the following criteria: samples with a genotyping call rate >98%, SNP genotypes with a call rate ≥98%, MAF > 0.01, Hardy-Weinberg Equilibrium (HWE) P value > 10*-6*, and Mendelian errors in <1% of the families. Imputation was performed on a genome-wide scale using a Sardinian sequence-based reference panel of 3514 individuals and the software Minimac^70^ on pre-phased genotypes^69^. After imputation, only markers with imputation quality (RSQR) > 0.3 for estimated minor allele frequency (MAF) ≥ 1% or RSQR > 0.6 for MAF < 1% were retained for association analyses^71^. To calculate the statistical power to find an effect of 0.59 standard deviation units, for a variant with a MAF of 0.05 (rs140884539) and an effect of 0.30 for a variant with a MAF of 0.21 (rs12323905), the tool G*Power v3.1.9.4 was used, considering the F test as the test family, the linear multiple regression as statistical test, a sample size of *N* = 999, and a type I error alpha of 5e-7.

### Genetic association and colocalisation analyses

#### Genome-wide association analysis

Genome-wide association analysis was carried out using the q.emmax (quantitative EMMAX–Efficient Mixed Model Association eXpedited) function included in EPACTS -3.2.6 (https://genome.sph.umich.edu/wiki/EPACTS). The method implemented in this software accounts for a wide range of sample structures, such as cryptic relatedness and population stratification, by applying a linear mixed model adjusted for a genomic-based kinship matrix obtained from quality-checked genotyped autosomal SNPs with MAF > 1%^69^. Urine neopterin levels were normalised by inverse-normal transformation and adjusted for sex and age as covariates. Variants located on the sex chromosomes were not tested. Because of the small sample size, variants with MAF < 1% were considered rare and not tested. MAFs reported for the European population (EUR) are from the 1000 Genome Project (https://www.internationalgenome.org/). The significance threshold for GWAS was 6.9×10^-09^, which was calculated by estimating the number of independent variants tested, as previously described^69^. Hardy–Weinberg equilibrium was considered and reported in the Supplementary Data 4.

#### Neopterin conditional analyses

Conditional analyses were performed in a region of +/-1Mb around the most associated variant, by adding it as a covariate to the association model adjusted for age and sex, as previously described for GWAS analyses.

#### Combined association analyses

Combined association analyses were performed to evaluate the marginal effects of multiple associated variants on the phenotype by including them in a regression model adjusted for sex and age; analyses were conducted using R software version 4.2.3 (https://cran.r-project.org/).

#### Variant pruning

Variant pruning was applied to estimate the number of independent variants at the *GCH1* gene locus using the option *--indep-pairwise 50 10 0.01* implemented in Plink-v1.09 (https://www.cog-genomics.org/plink/1.9/).

#### Colocalisation analysis

Colocalisation analysis was performed using Coloc v5.2.-3 package^72^ with default settings and excluding SNPs with MAF < 1%. Traits were considered as colocalising if the posterior probability of colocalisation (coloc_P(H4)) was greater than or equal to 0.8; uncertain if P(H4) ranged between 0.8 and 0.2, and not colocalising if below 0.2.

#### Fine mapping

Fine mapping was applied to the GWAS summary statistics to estimate the 95% credible set, the minimum set of variants including the causal variant at the locus, with a 95% summed posterior probability. Credible sets were obtained using the *FINEMAP software v 1.4.2*^73^ using the option --*sss* under the assumption of a maximum of five causal variants (--n-causal-snps 5).

#### Variants prioritisation

Variants prioritisation was based on fine mapping posterior probability, on the functional role of variants as established by the Variant Effect Predictor – VEP (https://www.ensembl.org/Tools/VEP) and on their known role in Parkinson’s disease.

### Expression and splicing quantitative trait loci analysis

SardiNIA eQTLs and sQTLs were previously described by Pala et al.^46^. We used the original eQTLs summary statistics and performed conditional analysis by the COJO module of GCTA (version 1.93)^74^.

To analyse the expression levels of individual *GCH1* isoforms in the SardiNIA dataset, we used Fragments Per Kilobase of exon per Million mapped reads (FPKM) values computed in Pala et al.^46^, for each transcript. The 213/250aa isoform ratio was calculated by dividing the FPKM of ENST00000536224.2 (213aa, a minor isoform) by that of ENST00000491895.2 (250aa, the canonical isoform). We observed that this ratio became disproportionately large in samples with low expression of the 250aa isoform. Therefore, we restricted the analysis to individuals in whom ENST00000491895.2 was expressed at FPKM > 1.

To test the association between either the individual isoforms or the 213aa/250aa ratio and the genetic variant rs11158026, we first regressed out the effects of sex, age, and age squared using a linear model. The residuals were then subjected to inverse normal transformation before genome-wide association testing by GEMMA^75^. Unlike the original QTL analysis in Pala et al.^46^, which included 606 individuals, the present analysis was conducted on 621 individuals, as we used an updated version of the SardiNIA genomic dataset that included a larger number of SardiNIA participants. Of these 621, 605 passed the FPKM > 1 threshold for 250aa (ENST00000491895.2) and were retained for the analysis.

### GCH1 in silico studies

The crystallographic structure of the 250aa isoform of GCH1, obtained from the Protein Data Bank (PDB ID: 1FB1**;** https://www.rcsb.org/structure/1FB1), was used to generate the dimer. The structure of the 213aa isoform was predicted by AlphaFold2 software^76^ based on the corresponding amino acid sequence downloaded from UniProt (P30793-2). The homodecameric complex and the mutated decameric form were constructed starting from the cryo-electron microscopy structure 6Z80 (https://www.rcsb.org/structure/6Z80)^77^. All structures were prepared using the Protein Preparation Wizard tool (Schrödinger Release 2024-3)^78^.

The stability of the GCH1 dimers and decamers was evaluated by molecular dynamics simulations by Desmond^79^ (Schrödinger Suite Release 2024-3) and the TIP3P solvent model^80^ was employed. They were placed in an orthorhombic water box, which extended 12.0 Å, and the box volumes were minimised and neutralised by adding ions (Na+ or Cl − ). The OPLS4 force field was chosen. Molecular dynamics simulations were conducted for a duration of 500 nsec for dimeric forms and 250nsec for decameric forms. The NPT ensemble was used with a constant temperature of 300.0 K using the Nosé–Hoover thermostat and a pressure of 1.01325 bar using the Martyna–Tobias–Klein barostat^81–83^. The trajectories obtained from the molecular dynamics simulations were analysed by calculating the binding free energy using the MMGBSA method, which allows for the estimation of free energy by accounting for both atomic interactions and solvent effects. The calculations were performed using the thermal_mmgbsa.py script integrated within Desmond^79^.

### Differentiation and Positive Selection Analyses

Two different measures were used to evaluate whether an associated variant can be considered under positive selection: the fixation index (Fst)^84^ to select a set of variants highly differentiated, and the integrated haplotype score (iHS) to test for positive selection^85^.

Fst, a frequency-based statistic, was used to evaluate the differentiation of a SNP allele between Sardinians and 32 worldwide populations from the 1000 Genome Project (https://www.internationalgenome.org/). Fst was calculated using the Weir-Cockerham formula implemented in *vcftools v.0.1.12b* (http://vcftools.sourceforge.net/).

In case of appreciable differentiation, positive selection can be evaluated using haplotype-based methods. iHS, which measures the amount of extended haplotype homozygosity (EHH) at a given SNP along the derived allele relative to the ancestral allele, was applied. EHH and iHS values were calculated using *selscan-v2.0.0* (https://github.com/szpiech/selscan).

Ancestral and derived alleles were established through the Variant Effect Predictor (VEP) tool (https://www.ensembl.org/info/docs/tools/vep/index.html). The significance of results was defined empirically using a “genomic percentile”, comparing the statistics for each tested variant to those of a set of variants with similar genomic characteristics (allele frequency in Sardinians, local recombination rate, and levels of background selection)^86^.

A significant iHS value is positive when the ancestral allele lies in a more extended haplotype than the derived allele. In contrast, it is negative when the derived allele lies in a more extended haplotype. A larger-than-average haplotype extension, combined with high frequency, means that the tested allele increased rapidly in frequency so that recombination could not break the LD around it; this indicates a positive selection effect on the variant.

### Regional genetic analysis of neurodegenerative molecules

Specific association analyses in the *GCH1* gene region (1751 variants) were carried out for Tau, pTau-181, amyloid beta 40 and 42, neurofilament light chain, and glial fibrillary acidic protein on up to 5216 volunteers. The association threshold was 4.76 × 10^-06^, calculated by dividing the nominal significance threshold of 0.05 by the number of traits (*n*.6) and independent variants (*n*.1751) tested simultaneously.

## Supplementary information

Supplementary information

Supplementary data

## Data Availability

The GWAS summary statistics have been deposited in the Figshare repository (https://figshare.com/) under the accession number 10.6084/m9.figshare.30639128.
