# Supplementary material for: Genetic co-regulation of neopterin and Parkinson’s disease
Source: NPJ Parkinsons Dis. 2026 Feb 10;12:69. doi: 10.1038/s41531-026-01279-x (PMC13002981; doi:10.1038/s41531-026-01279-x)
Supplement: Supplementary file 1 — Supplementary information [file 41531_2026_1279_MOESM1_ESM.pdf]

Supplementary Information

Supplementary Information includes Supplementary Figures, Supplementary Data, and Supplementary Results.

Supplementary Figures

SardiNIA

r² [D']	1° neopterin var rs140884539:C>T	2° neopterin var rs12323905:T>C	Parkinson's var rs11158026:C>T
1° neopterin var rs140884539:C>T	1 [1] CC/TT		
2° neopterin var rs12323905:T>C	0.21 [1] TC/CT	1 [1] CC/TT	
Parkinson's var *rs11158026:C>T*	0.22 [0.97] CC/TT	0.92 [1] CT/TC	1 [1] CC/TT

1000G EUR

r² [D']	1° neopterin var rs140884539:C>T	2° neopterin var rs12323905:T>C	Parkinson's var rs11158026:C>T
1° neopterin var rs140884539:C>T	1 [1] CC/TT		
2° neopterin var rs12323905:T>C	0.21 [1] TC/CT	1 [1] CC/TT	
Parkinson's var rs11158026:C>T	0.21 [1] CC/TT	0.96 [0.995] CT/TC	1 [1] CC/TT

1000G EAS

r² [D']	1° neopterin var rs140884539:C>T	2° neopterin var rs12323905:T>C	Parkinson's var rs11158026:C>T
1° neopterin var rs140884539:C>T	1 [1] CC/TT		
2° neopterin var rs12323905:T>C	0.58 [1] TC/CT	1 [1] CC/TT	
Parkinson's var *rs11158026:C>T*	0.56 [0.96] CC/TT	0.97 [1] CT/TC	1 [1] CC/TT

1000G Africa

r² [D']	1° neopterin var rs140884539:C>T	2° neopterin var rs12323905:T>C	Parkinson's var rs11158026:C>T
1° neopterin var rs140884539:C>T	1 [1] CC/TT		
2° neopterin var rs12323905:T>C	0.32 [0.994] TC/CT	1 [1] CC/TT	
Parkinson's var *rs11158026:C>T*	0.32 [0.994] CC/TT	0.98 [0.99] CT/TC	1 [1] CC/TT

Supplementary Figure 1. Linkage disequilibrium of neopterin- and Parkinson's disease-associated variants. Linkage disequilibrium expressed as r^2 and D' is evaluated in the SardiNIA cohort, Europeans (EUR), East Asia (EAS), and Africa (AFR). Data for the latter three populations are from the 1000 Genomes Project (https://www.internationalgenome.org/).

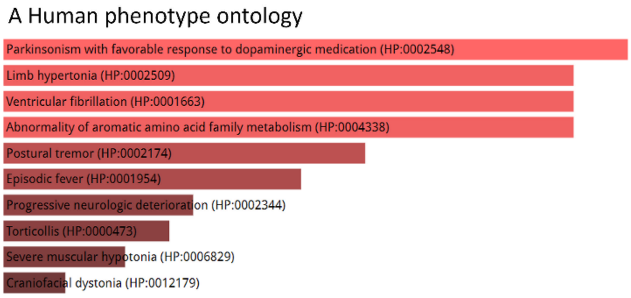

Index	Name	P-value	Adjusted p-value	Odds Ratio	Combined score
1	Parkinsonism with favorable response to dopaminergic medication (HP:0002548)	0.01564	0.1216	73.37	305.06
2	Limb hypertonia (HP:0002509)	0.01737	0.1216	65.22	264.33
3	Ventricular fibrillation (HP:0001663)	0.01737	0.1216	65.22	264.33
4	Abnormality of aromatic amino acid family metabolism (HP:0004338)	0.01737	0.1216	65.22	264.33
5	Postural tremor (HP:0002174)	0.02594	0.1262	41.91	153.07
6	Episodic fever (HP:0001954)	0.02935	0.1262	36.67	129.39
7	Progressive neurologic deterioration (HP:0002344)	0.03613	0.1262	29.33	97.40
8	Torticollis (HP:0000473)	0.03782	0.1262	27.93	91.48
9	Severe muscular hypotonia (HP:0006829)	0.04119	0.1262	25.50	81.34
10	Craniofacial dystonia (HP:0012179)	0.04622	0.1262	22.56	69.34

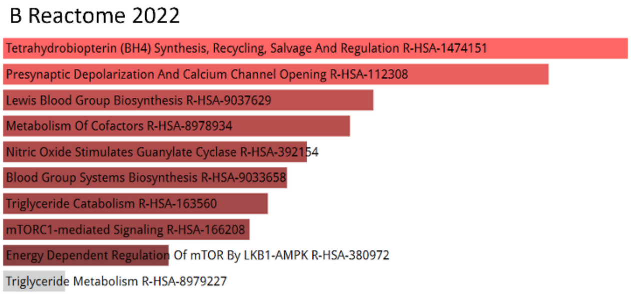

Index	Name	P-value	Adjusted p-value	Odds Ratio	Combined score
1	Tetrahydrobiopterin (BH4) Synthesis, Recycling, Salvage And Regulation R-HSA-1474151	0.01737	0.3728	65.22	264.33
2	Presynaptic Depolarization And Calcium Channel Opening R-HSA-112308	0.02080	0.3728	53.35	206.61
3	Lewis Blood Group Biosynthesis R-HSA-9037629	0.03105	0.3728	34.51	119.83
4	Metabolism Of Cofactors R-HSA-8978934	0.03275	0.3728	32.59	111.44
5	Nitric Oxide Stimulates Guanylate Cyclase R-HSA-392154	0.03613	0.3728	29.33	97.40
6	Blood Group Systems Biosynthesis R-HSA-9033658	0.03782	0.3728	27.93	91.48
7	Triglyceride Catabolism R-HSA-163560	0.03951	0.3728	26.66	86.15
8	mTORC1-mediated Signaling R-HSA-166208	0.04119	0.3728	25.50	81.34
9	Energy Dependent Regulation Of mTOR By LKB1-AMPK R-HSA-380972	0.04956	0.3728	20.94	62.92
10	Triglyceride Metabolism R-HSA-8979227	0.06281	0.3728	16.28	45.06

Supplementary Figure 2. Enrichr pathway enrichment of neopterin. The enriched pathways were obtained by loading genes from the neopterin GWAS (Supplementary Table 4) in the Enrichr database. The first top 10 enriched pathways are indicated.

Supplementary Data

Supplementary Data 1. Kits used to measure haematochemical parameters and neurodegeneration molecules.

Supplementary Data 2. Summary statistics and correlation of neopterin with haematochemical parameters, and neurodegeneration molecules.

Supplementary Data 3. Heritability estimation of neopterin.

Supplementary Data 4. Results of neopterin GWAS.

Supplementary Data 5. Credible set 1.

Supplementary Data 6. Credible set 2.

Supplementary Data 7. Joint analysis.

Supplementary Data 8. Summary statistics of eQTL SardiNIA data.

Supplementary Data 9. *GCH1* eQTL data from GTEX.

Supplementary Data 10. *GCH1* sQTL data from GTEX.

Supplementary Data 11. Colocalisation among neopterin, Parkinson's disease, *GCH1* eQTL, and sQTL signals (extended).

Supplementary Results

Differentiation and selection of the neopterin signals

The variants associated with neopterin, rs140884539-C and rs12323905-T, are characterised by differentiation worldwide, with Sardinians having the highest frequencies (Supplementary Fig. 3). In populations of African origin, we observed a strong indication of differentiation of both variants with respect to Sardinians, especially in Mende from Sierra Leone (MSL) and Americans of African ancestry (ASW) populations (Supplementary Table 1). Variant rs140884539 was also significantly differentiated in Sardinia with respect to the South Asian population. We thus tested positive selective pressure in Sardinia using the integrated haplotype score (iHS) test, but we did not detect any indication of positive selection (Supplementary Fig. 4, Supplementary Table 2).

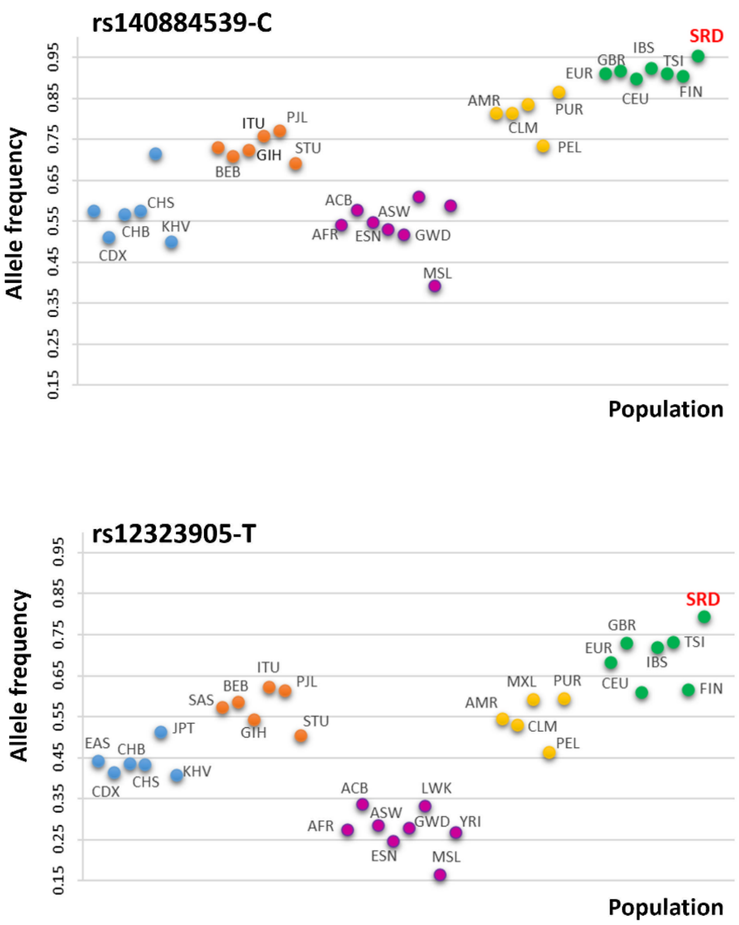

Population	Description
EAS	East Asian
CDX	Chinese Dai in Xishuangbanna, China
CHB	Han Chinese in Beijing, China
CHS	Southern Han Chinese
JPT	Japanese in Tokyo, Japan
KHV	Kinh in Ho Chi Minh City, Vietnam
SAS	South Asian
BEB	Bengali from Bangladesh
GIH	Gujarati Indian from Houston, Texas
ITU	Indian Telugu from the UK
PJL	Punjabi from Lahore, Pakistan
STU	Sri Lankan Tamil from the UK
AFR	African
ACB	African Caribbeans in Barbados
ASW	Americans of African Ancestry in SW USA
ESN	Esan in Nigeria
GWD	Gambian in Western Divisions in the Gambia
LWK	Luhya in Webuye, Kenya
MSL	Mende in Sierra Leone
YRI	Yoruba in Ibadan, Nigeria
AMR	Ad Mixed American
CLM	Colombians from Medellin, Colombia
MXL	Mexican Ancestry from Los Angeles USA
PEL	Peruvians from Lima, Peru
PUR	Puerto Ricans from Puerto Rico
EUR	European
GBR	British in England and Scotland
CEU	Utah Residents (CEPH) with Northern and Western European Ancestry
IBS	Iberian Population in Spain
TSI	Toscani in Italia
FIN	Finnish in Finland
SRD	Sardinia

Supplementary Figure 3. Allele frequency of rs140884539-C and rs12323905-T. Allelic frequencies of all populations, except Sardinians (SRD, in red), refer to 1000 Genome Phase 3. Population acronyms are legend-coded.

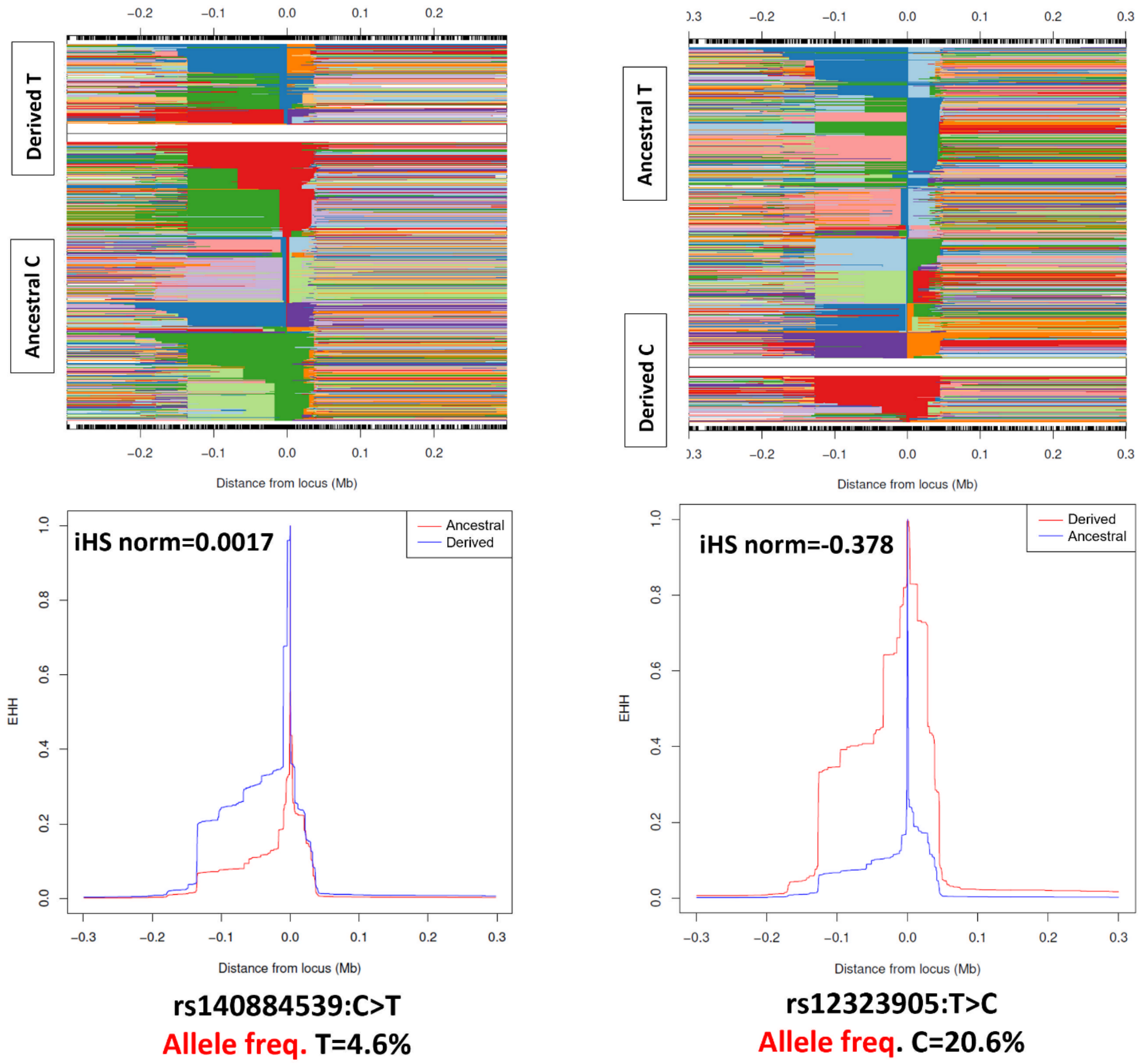

Supplementary Figure 4. Extended haplotype homozygosity around rs140884539 and rs12323905 in the SardiNIA cohort. Extended Haplotype Homozygosity (EHH) decay as a function of the distance around the ancestral and derived alleles for rs140884539 and rs12323905 variants. In the upper panels, horizontal lines represent haplotypes; haplotypes with the same color carry identical alleles. In the lower panels, decay lines of haplotype homozygosity are shown.

		rs140884539				rs12323905			
Population	*Subpopulation*	*Marker*	*N of matching variants*	*Fst*	*Genomic percentile*	*Marker*	*N of matching*	*Fst*	*Genomic percentile*
**EUR**	**EUR**	**14:55341903**	**2277**	**0.0058**	**62%**	**14:55335468**	**535**	**0.0174**	**81%**
EUR	EURnoTSI	14:55341903	3537	0.0036	45%	14:55335468	820	0.0072	49%
EUR	FIN	14:55341903	2485	0.0083	56%	14:55335468	624	0.0555	85%
EUR	GBR	14:55341903	2153	0.0014	43%	14:55335468	518	0.0005	34%
EUR	IBS	14:55341903	3492	-0.0023	33%	14:55335468	821	-0.0040	20%
EUR	TSI	14:55341903	2142	0.0046	67%	14:55335468	506	0.0004	43%
**AFR**	**AFR**	**14:55341903**	**991**	**0.4132**	**93%**	**14:55335468**	**647**	**0.3936**	**94%**
AFR	ACB	14:55341903	1849	0.2986	95%	14:55335468	812	0.2712	90%
AFR	ASW	14:55341903	1903	0.3621	97%	14:55335468	812	0.3347	96%
AFR	ESN	14:55341903	1105	0.3467	92%	14:55335468	682	0.3848	92%
AFR	GWD	14:55341903	1118	0.3512	93%	14:55335468	705	0.3441	91%
AFR	LWK	14:55341903	1263	0.2623	90%	14:55335468	744	0.2770	88%
AFR	MSL	14:55341903	1094	0.5051	96%	14:55335468	688	0.4958	95%
AFR	YRI	14:55341903	1077	0.2810	88%	14:55335468	677	0.3571	91%
**AMR**	**AMR**	**14:55341903**	**2373**	**0.0967**	**94%**	**14:55335468**	**587**	**0.1113**	**93%**
AMR	CLM	14:55341903	3345	0.0703	93%	14:55335468	820	0.0819	87%
AMR	MXL	14:55341903	2862	0.0565	86%	14:55335468	820	0.0406	58%
AMR	PEL	14:55341903	2302	0.1451	90%	14:55335468	820	0.1384	71%
AMR	PUR	14:55341903	3247	0.0296	80%	14:55335468	820	0.0384	79%
**ASIA**	**ASIA**	**14:55341903**	**1766**	**0.1646**	**95%**	**14:55335468**	**789**	**0.0950**	**79%**
**EAS**	**EAS**	**14:55341903**	**985**	**0.3934**	**94%**	**14:55335468**	**627**	**0.2106**	**88%**
EAS	CDX	14:55341903	1058	0.3716	96%	14:55335468	699	0.1884	79%
EAS	CHB	14:55341903	1001	0.3046	94%	14:55335468	683	0.1644	78%
EAS	CHS	14:55341903	992	0.2945	93%	14:55335468	680	0.1679	77%
EAS	JPT	14:55341903	1004	0.1562	81%	14:55335468	679	0.0943	63%
EAS	KHV	14:55341903	1099	0.3791	96%	14:55335468	715	0.1932	80%
**SAS**	**SAS**	**14:55341903**	**1754**	**0.1956**	**97%**	**14:55335468**	**629**	**0.0862**	**82%**
SAS	BEB	14:55341903	2232	0.1704	97%	14:55335468	818	0.0444	56%
SAS	ITU	14:55341903	1955	0.1174	93%	14:55335468	807	0.0248	46%
SAS	PJL	14:55341903	2580	0.1076	95%	14:55335468	820	0.0281	54%
SAS	STU	14:55341903	1956	0.1813	97%	14:55335468	810	0.1024	77%
SAS	GIH	14:55341903	2111	0.1503	96%	14:55335468	818	0.0723	72%

Supplementary Table 1. Differentiation analysis by Fixation Index.
Fst=Fixation Index

rs140884539

Population	*Sub_Population*	*Marker*	*allele 1-T, allele 0-C*	*1_freq T*	*ihh_1 T*	*ihh_0 C*	*iHS*	*iHS_norm*	*Genomic percentile*
**EUR**	**EUR**	**14:55341903**	**T-C**	**0.0885**	**0.0347**	**0.0036**	**2.2656**	**0.9402**	45%
EUR	CEU	14:55341903	T-C	0.1010	0.0372	0.0021	2.8803	1.7510	79%
EUR	EURnoTSI	14:55341903	T-C	0.0884	0.0315	0.0031	2.3153	0.9556	47%
EUR	FIN	14:55341903	T-C	0.0960	0.0257	0.0019	2.6235	0.9680	54%
EUR	GBR	14:55341903	T-C	0.0824	0.0284	0.0032	2.1966	0.7739	43%
EUR	GBR_FIN	14:55341903	T-C	0.0895	0.0118	0.0013	2.2019	0.8919	50%
EUR	IBS	14:55341903	T-C	0.0748	0.0231	0.0048	1.5742	0.0296	1%
**EUR**	**SARD**	**14:55341903**	**T-C**	**0.0599**	**0.0227**	**0.0042**	**1.6790**	**0.0017**	**19%**
EUR	TSI	14:55341903	T-C	0.0888	0.0422	0.0036	2.4687	1.1887	59%
**AFR**	**AFR**	**14:55341903**	**T-C**	**0.4592**	**0.0008**	**0.0009**	**-0.0856**	**-0.2072**	15%
AFR	ACB	14:55341903	T-C	0.4219	0.0010	0.0014	-0.3418	-0.5796	38%
AFR	ASW	14:55341903	T-C	0.4508	0.0006	0.0016	-0.9646	-0.9629	58%
AFR	ESN	14:55341903	T-C	0.4697	0.0014	0.0016	-0.1310	-0.2603	17%
AFR	GWD	14:55341903	T-C	0.4823	0.0023	0.0016	0.3837	0.3329	21%
AFR	LWK	14:55341903	T-C	0.3889	0.0006	0.0013	-0.8162	-1.2671	71%
AFR	MSL	14:55341903	T-C	0.6059	0.0008	0.0015	-0.6636	-0.1066	6%
AFR	YRI	14:55341903	T-C	0.4120	0.0033	0.0013	0.9366	0.5257	34%
**AMR**	**AMR**	**14:55341903**	**T-C**	**0.1859**	**0.0096**	**0.0015**	**1.8345**	**0.9901**	52%
AMR	CLM	14:55341903	T-C	0.1862	0.0069	0.0025	0.9933	-0.0364	2%
AMR	MXL	14:55341903	T-C	0.1641	0.0253	0.0034	2.0209	1.0051	57%
AMR	PEL	14:55341903	T-C	0.2647	0.0410	0.0036	2.4273	1.7681	88%
AMR	PUR	14:55341903	T-C	0.1346	0.0049	0.0032	0.4188	-0.8152	43%
**ASIA**	**ASIA**	**14:55341903**	**T-C**	**0.3474**	**0.0053**	**0.0052**	**0.0237**	**-0.4131**	25%
**EAS**	**EAS**	**14:55341903**	**T-C**	**0.4236**	**0.0056**	**0.0054**	**0.0284**	**-0.2661**	15%
EAS	CDX	14:55341903	T-C	0.4892	0.0065	0.0055	0.1744	0.0243	1%
EAS	CHB	14:55341903	T-C	0.4320	0.0092	0.0072	0.2410	0.0377	2%
EAS	CHS	14:55341903	T-C	0.4238	0.0093	0.0139	-0.4063	-0.8524	54%
EAS	JPT	14:55341903	T-C	0.2837	0.0109	0.0071	0.4248	-0.1534	11%
EAS	KHV	14:55341903	T-C	0.5000	0.0098	0.0065	0.4030	0.3786	25%
**SAS**	**SAS**	**14:55341903**	**T-C**	**0.2689**	**0.0045**	**0.0031**	**0.3590**	**-0.2881**	18%
SAS	BEB	14:55341903	T-C	0.2907	0.0070	0.0064	0.0943	-0.5671	35%
SAS	ITU	14:55341903	T-C	0.2402	0.0059	0.0073	-0.2119	-1.1987	65%
SAS	PJL	14:55341903	T-C	0.2292	0.0107	0.0060	0.5753	-0.2551	16%
SAS	STU	14:55341903	T-C	0.3088	0.0087	0.0064	0.3151	-0.3171	19%

rs12323905

Population	Sub_Population	Marker	allele 1-C, allele 0-T	1_freq C	ihh_1 C	ihh_0 T	iHS	iHS_norm	Genomic percentile
**EUR**	**EUR**	**14:55335468**	**C-T**	**0.3171**	**0.0115**	**0.0078**	**0.3919**	**-0.1991**	12%
EUR	CEU	14:55335468	C-T	0.3889	0.0070	0.0068	0.0238	-0.3513	22%
EUR	EURnoTSI	14:55335468	C-T	0.3308	0.0095	0.0073	0.2685	-0.2578	16%
EUR	FIN	14:55335468	C-T	0.3838	0.0065	0.0044	0.4044	0.0388	2%
EUR	GBR	14:55335468	C-T	0.2692	0.0085	0.0067	0.2310	-0.5791	35%
EUR	GBR_FIN	14:55335468	C-T	0.3289	0.0022	0.0032	-0.3662	-1.0292	62%
EUR	IBS	14:55335468	C-T	0.2804	0.0132	0.0106	0.2221	-0.4861	29%
**EUR**	**SARD**	**14:55335468**	**C-T**	**0.2340**	**0.0132**	**0.0080**	**0.5041**	**-0.3783**	**30%**
EUR	TSI	14:55335468	C-T	0.2664	0.0107	0.0076	0.3349	-0.4449	28%
**AFR**	**AFR**	**14:55335468**	**C-T**	**0.7247**	**0.0004**	**0.0051**	**-2.6668**	**-1.4954**	74%
AFR	ACB	14:55335468	C-T	0.6615	0.0005	0.0045	-2.1157	-1.1923	66%
AFR	ASW	14:55335468	C-T	0.7131	0.0004	0.0072	-2.8922	-1.6576	80%
AFR	ESN	14:55335468	C-T	0.7525	0.0009	0.0077	-2.1592	-0.7124	44%
AFR	GWD	14:55335468	C-T	0.7212	0.0011	0.0061	-1.7334	-0.6076	40%
AFR	LWK	14:55335468	C-T	0.6667	0.0003	0.0062	-2.9200	-1.8592	85%
AFR	MSL	14:55335468	C-T	0.8353	0.0004	0.0089	-3.1524	-1.2496	70%
AFR	YRI	14:55335468	C-T	0.7315	0.0009	0.0076	-2.1583	-0.8188	51%
**AMR**	**AMR**	**14:55335468**	**C-T**	**0.4539**	**0.0017**	**0.0035**	**-0.7246**	**-0.9158**	55%
AMR	CLM	14:55335468	C-T	0.4681	0.0049	0.0057	-0.1632	-0.3633	22%
AMR	MXL	14:55335468	C-T	0.4063	0.0072	0.0070	0.0189	-0.3384	28%
AMR	PEL	14:55335468	C-T	0.5353	0.0108	0.0074	0.3803	0.2470	18%
AMR	PUR	14:55335468	C-T	0.4038	0.0061	0.0070	-0.1407	-0.4314	27%
**ASIA**	**ASIA**	**14:55335468**	**C-T**	**0.4930**	**0.0018**	**0.0089**	**-1.5976**	**-1.8773**	88%
**EAS**	**EAS**	**14:55335468**	**C-T**	**0.5575**	**0.0029**	**0.0089**	**-1.1206**	**-1.2766**	74%
EAS	CDX	14:55335468	C-T	0.5860	0.0050	0.0086	-0.5444	-0.6290	40%
EAS	CHB	14:55335468	C-T	0.5631	0.0059	0.0135	-0.8346	-0.9476	63%
EAS	CHS	14:55335468	C-T	0.5667	0.0056	0.0244	-1.4649	-1.7177	85%
EAS	JPT	14:55335468	C-T	0.4856	0.0057	0.0135	-0.8707	-1.2640	73%
EAS	KHV	14:55335468	C-T	0.5909	0.0074	0.0099	-0.2927	-0.2672	20%
**SAS**	**SAS**	**14:55335468**	**C-T**	**0.4264**	**0.0015**	**0.0070**	**-1.5224**	**-2.0117**	88%
SAS	BEB	14:55335468	C-T	0.4128	0.0044	0.0092	-0.7485	-1.1778	67%
SAS	ITU	14:55335468	C-T	0.3775	0.0038	0.0142	-1.3110	-2.0124	90%
SAS	PJL	14:55335468	C-T	0.3854	0.0060	0.0107	-0.5740	-1.0955	64%
SAS	STU	14:55335468	C-T	0.4951	0.0055	0.0125	-0.8281	-1.0199	62%

Supplementary Table 2. Selection analysis by integrated Haplotype Score for rs140884539 and rs12323905. Allele 1 refers to the derived allele, allele 0 to the ancestral allele. ihh=integrated Haplotype Homozygosity, iHS= integrated Haplotype Score.

Genetic correlation of neopterin with neurodegenerative markers

Because *GCH1* is involved in neurotransmitter synthesis and neurodegenerative disorders,[87] we used data from 5204 SardiNIA volunteers to test whether variants in the *GCH1* gene were associated with six circulating molecules, namely Tau, phophoTau-181 (pTau-181), amyloid beta 40 and 42 (Aβ40 and Aβ42), neurofilament light chain (Nf-L), and glial fibrillary acidic protein (GFAP), which are markers of neurodegeneration, including Parkinson's disease.[18-21] A regional association study of the six neurodegeneration-related molecules with SNPs in the *GCH1* region detected no significant genetic association (Supplementary Fig. 5).

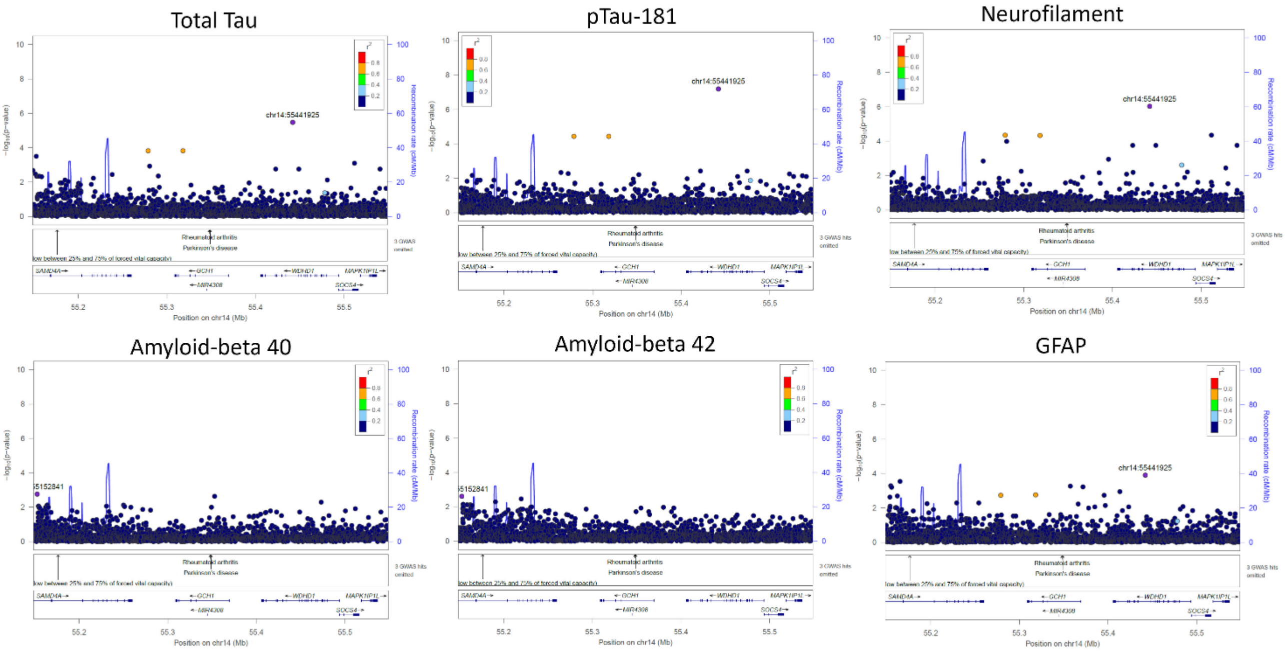

Supplementary Figure 5. Neurodegeneration molecule association plots of the *GCH1* region. The description of the axes is as in Fig. 3A.

Supplementary References

87. Gupta, P., Kumar, R. GTP cyclohydroxylase1 (GCH1): Role in neurodegenerative diseases. *Gene*. **888**, 147749 (2023).
